# An Adolescent Football Player With Persistent Headaches Following a Concussion: A Case of Primary Arachnoid Cyst

**DOI:** 10.7759/cureus.88183

**Published:** 2025-07-17

**Authors:** Jonathan R Guin, Ryan Moran, Earl R Stewart, Brett C Bentley

**Affiliations:** 1 Family Medicine, University of Alabama, Tuscaloosa, USA; 2 Athletic Training, University of Alabama, Tuscaloosa, USA

**Keywords:** brain concussion, congenital arachnoid cyst, management of arachnoid cyst, pediatric sports medicine, post-concussion headache

## Abstract

Post-concussion syndrome (PCS) is a common sequela of mild traumatic brain injury in adolescent athletes, typically resolving within weeks. However, persistent or atypical symptoms warrant further investigation to exclude structural pathology. Arachnoid cysts, though often incidental, can become symptomatic following trauma and may mimic or exacerbate PCS. A previously healthy adolescent American football player presented with persistent headaches and cognitive symptoms 14 weeks after a sports-related concussion. Despite completing a return-to-play protocol, his symptoms worsened. A brain MRI revealed a large left-sided arachnoid cyst with a 5 mm midline shift, consistent with a primary arachnoid cyst. The patient was admitted to the medical unit of the pediatric hospital for monitoring on the medical floor with 48 hours of observation and repeat imaging to verify that the MRI findings were not worsening. Serial imaging over three months demonstrated regression of the cyst and resolution of mass effect. He remained asymptomatic at follow-up but was permanently restricted from contact sports.

This case underscores the importance of considering structural brain lesions in athletes with prolonged or atypical post-concussive symptoms. MRI played a critical role in identifying a potentially life-threatening condition that mimicked PCS. Conservative management may be appropriate in select cases of arachnoid cysts with favorable clinical and radiographic progression. Surgical management may be necessary in cases of increased intracranial pressure, neurologic deficit, or radiographic progression.

## Introduction

Concussion is a common injury in adolescent athletes, particularly in contact sports such as American football. While most cases resolve within 7-10 days, some individuals experience persistent symptoms lasting weeks to months, referred to as post-concussion syndrome (PCS) [1]. According to Diagnostic and Statistical Manual of Mental Disorders, Fourth Edition (DSM-IV), PCS is diagnosed in patients with cognitive deficit in attention or memory and at least three of the following symptoms present for at least three months: fatigue, sleep disturbance, headache, dizziness, irritability, affective disturbance, apathy, or personality changes [2]. The symptoms must have also worsened after injury, impact social function, and cannot be better explained by dementia or another cause of symptoms [2]. Of note, in Diagnostic and Statistical Manual of Mental Disorders, Fifth Edition (DSM-V), PCS is controversially revised to either major or mild neurocognitive disorder due to traumatic brain injury. Although rare, structural brain abnormalities - such as arachnoid cysts - can mimic or exacerbate post-concussive symptoms and have severe consequences [3]. Arachnoid cysts are cerebrospinal fluid-filled sacs arising from duplication of the arachnoid membrane during development and are often discovered incidentally in children undergoing imaging after trauma [4,5]. Secondary arachnoid cysts may result from trauma, hemorrhage, or infection [6]. While typically asymptomatic, rupture - spontaneous or trauma-induced - can lead to mass effect, intracranial hypertension, or hemorrhage [7]. Approximately 75% of ruptures are reported following trauma [8]. Males and individuals under 18 years appear to be at higher risk, possibly due to both increased prevalence and greater exposure to contact injuries [1]. When present in the context of head injury, symptomatic arachnoid cysts remain rare but clinically significant [9]. This case describes a high school football player whose persistent headaches post-concussion revealed a primary arachnoid cyst causing midline shift without gross neurologic abnormality. It highlights the importance of advanced imaging to evaluate for structural pathology in post-concussion syndrome.

## Case presentation

Initial injury and recovery

An adolescent high school American football player with a past medical history only significant for attention-deficit/hyperactivity disorder (ADHD) suffered a concussion in late summer after sustaining a hard tackle. There was no loss of consciousness during the event. The athlete was able to continue practicing initially; however, he was evaluated by the athletic trainer after practice and was told he could no longer participate and needed to be assessed by a physician for a likely concussion. The patient was seen by an outside primary care provider and diagnosed with a concussion. As part of the concussion protocol, he was not allowed to participate in football. His symptoms improved over the next three weeks, and he was cleared to begin the return-to-play protocol. He completed the return to play protocol one week later and then resumed full football participation. He was then able to complete the remainder of the football season.

New injury and follow-up

**Figure 1:**
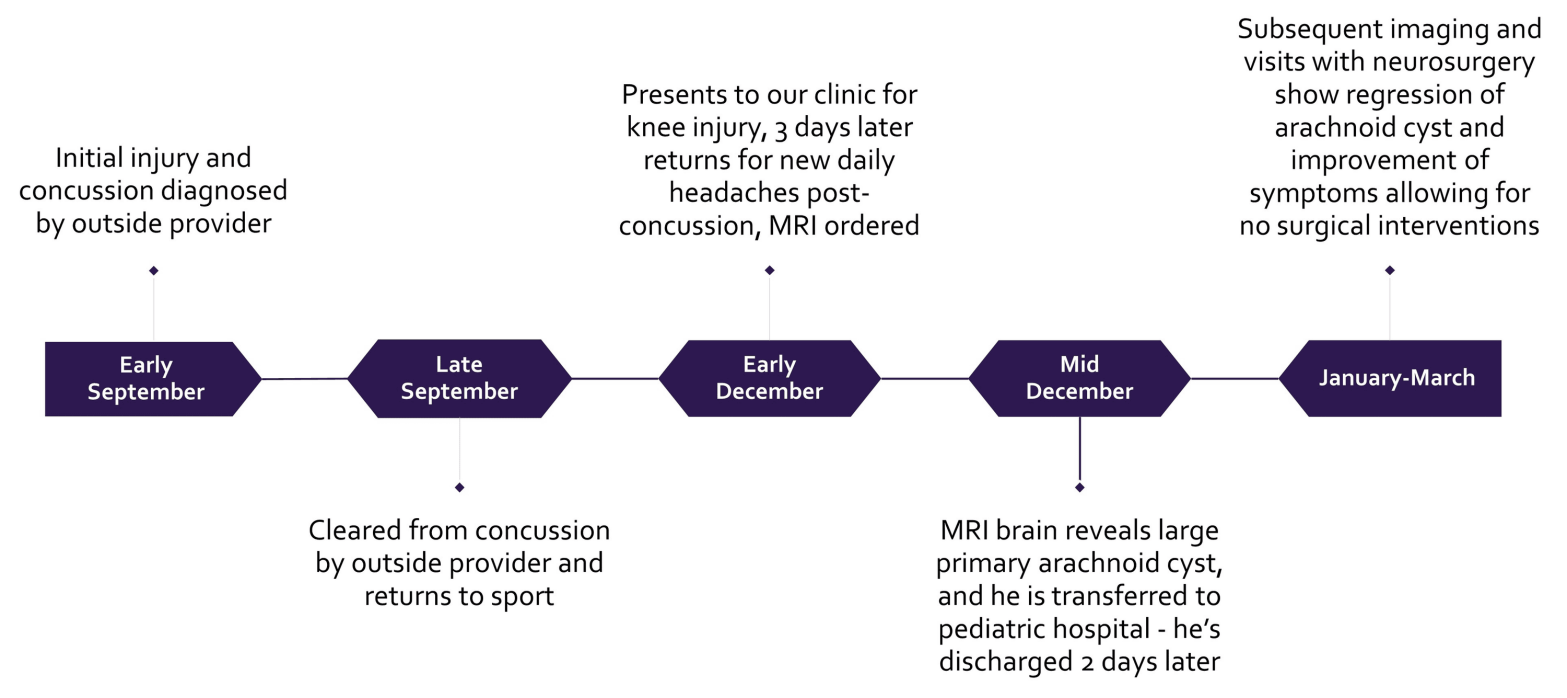
Timeline of case presentation including initial injury, diagnosis, and management from early September to March of the following year.

Fourteen weeks after his concussion, he presented to our clinic for evaluation of a right knee injury from three days prior. On his knee examination, he had a joint effusion and a positive medial McMurray’s test, prompting an MRI order for suspected medial meniscal pathology. The day after the visit for his knee injury, his mother contacted our clinic to report that he had also been experiencing new daily headaches since his concussion 14 weeks prior, which she had forgotten to mention during the visit for his knee injury, and she requested to return to the clinic for further evaluation for his post-concussion headaches. The patient returned three days later to discuss his daily headaches. On review of systems and completion of the SCAT5 symptom checklist (symptoms are rated from "0-6" where "0" is "none" and "6" is "severe"), he endorsed the following symptoms and severity scores: headache (3), pressure in the head (4), sensitivity to light (2), sensitivity to noise (2), feeling like "in a fog" (2), "don't feel right" (1), difficulty concentrating (3), fatigue (3), irritability (3), and trouble falling asleep (3). His total symptom score was 10 out of 22, with a severity score of 26 out of 132. We did not have access to the record from his initial visit with the outside provider who diagnosed the concussion, but he did self-report many of the same symptoms at this visit, just currently less severe than at the time of the concussion. His neurological exam at our visit, including cranial nerve assessment, mental status, motor strength, sensory function, and balance testing, was within normal limits. Notably, he had recently changed stimulant medications for ADHD and was concerned this could be contributing to his symptoms. A diagnosis of post-concussion syndrome was made, and a brain MRI was ordered to evaluate for structural pathology. The MRI was obtained one week later at an outpatient imaging center. Figure 1 displays the chronological sequence of events in the case presentation.

Neuroimaging and findings

**Figure 2:**
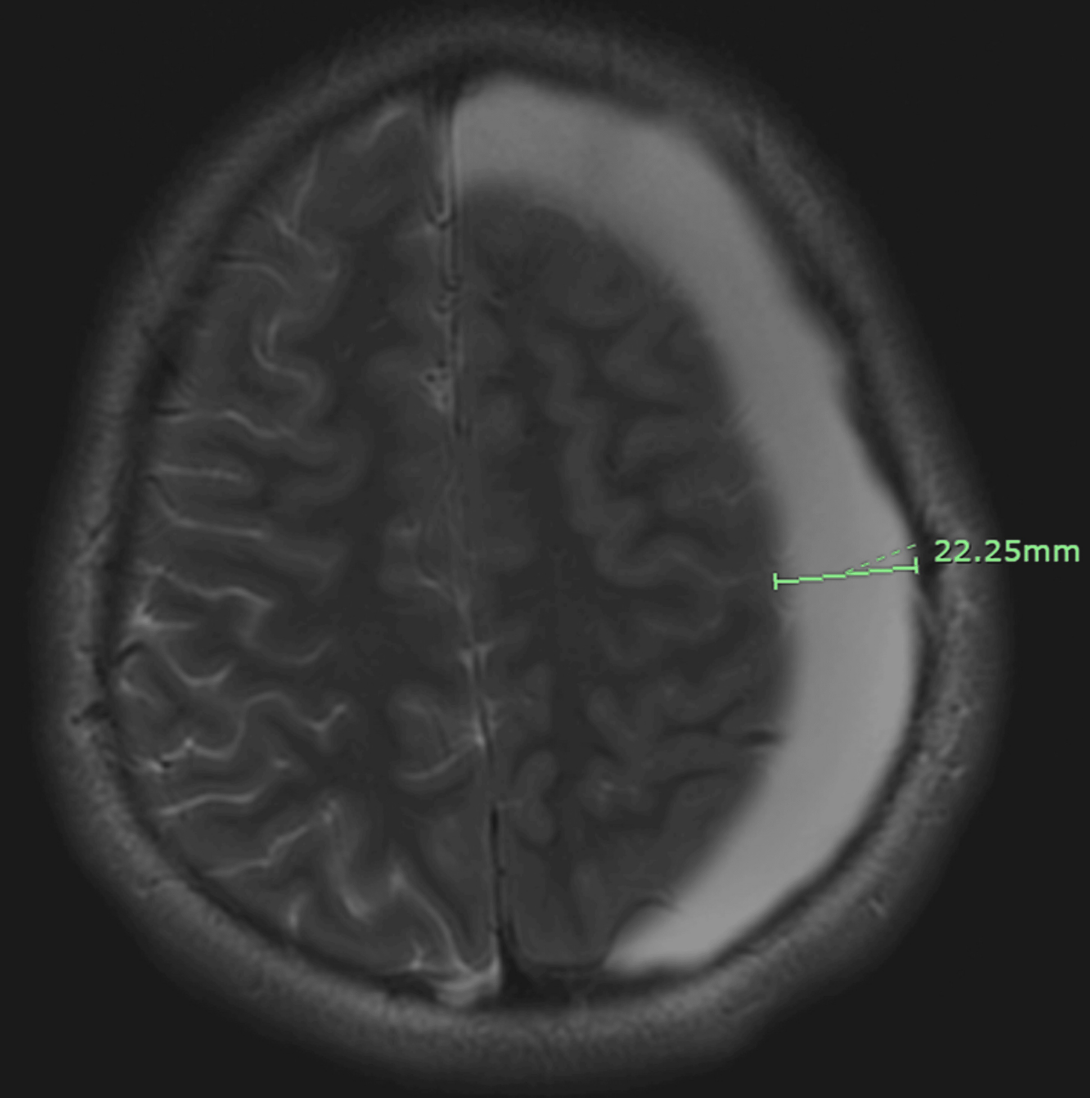
Axial T2-weighted brain MRI without contrast using the PROPELLER technique demonstrating 22.25 mm left temporal arachnoid cyst causing sulcal effacement. PROPELLER technique: Periodically Rotated Overlapping Parallel Lines with Enhanced Reconstruction

**Figure 3:**
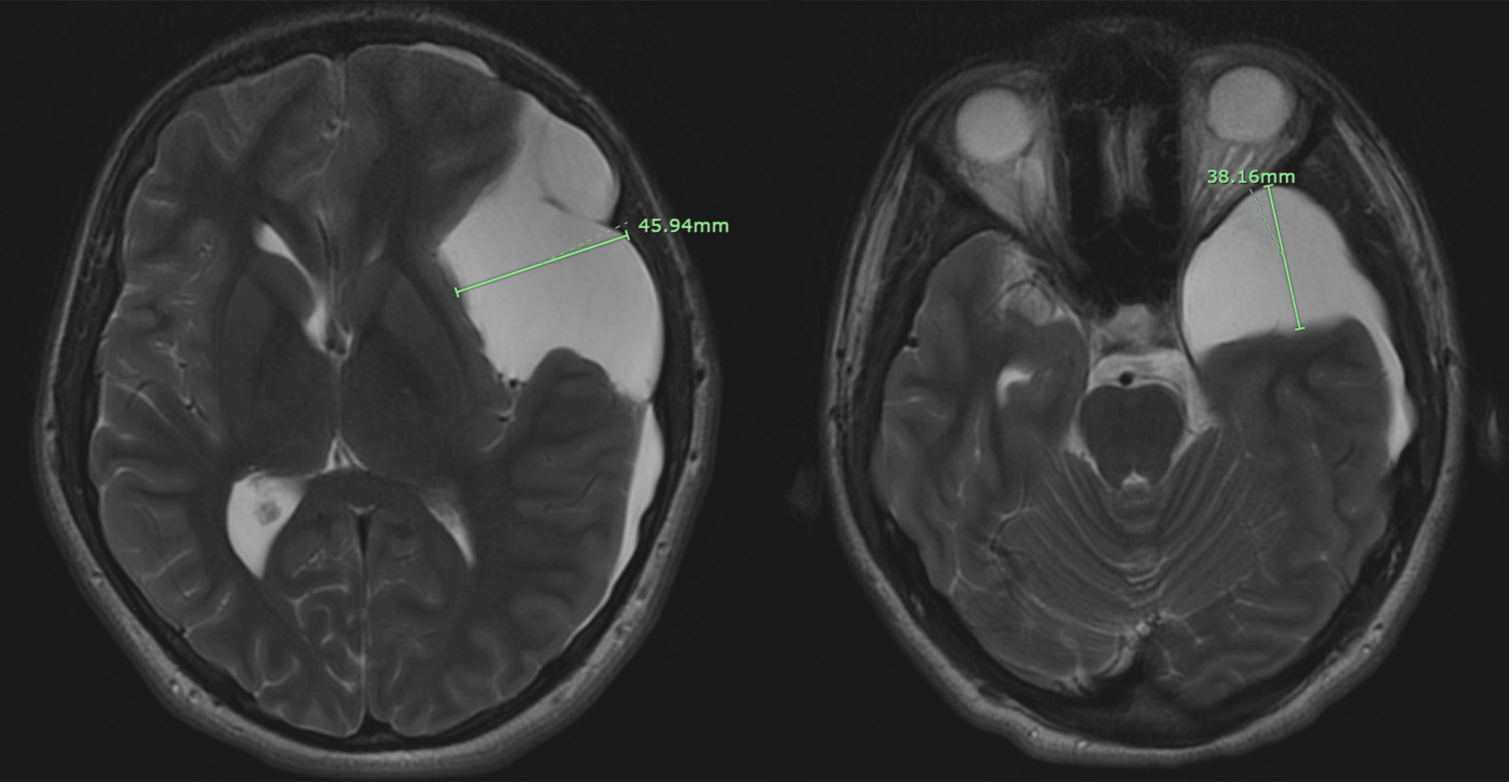
Axial T2-weighted brain MRI without contrast, using the PROPELLER technique, demonstrates a left cerebral hemisphere arachnoid cyst measuring 45.94 mm (left to right) × 38.16 mm, located caudally at the level of the orbits within the cranium, with partial compression of the left lateral ventricle. PROPELLER technique: Periodically Rotated Overlapping Parallel Lines with Enhanced Reconstruction

Brain MRI without contrast revealed a large left-sided extra-axial fluid collection consistent with a large arachnoid cyst versus chronic subdural hematoma measuring 22.25 × 45.94 × 38.16 mm (Figures 2, 3).

**Figure 4:**
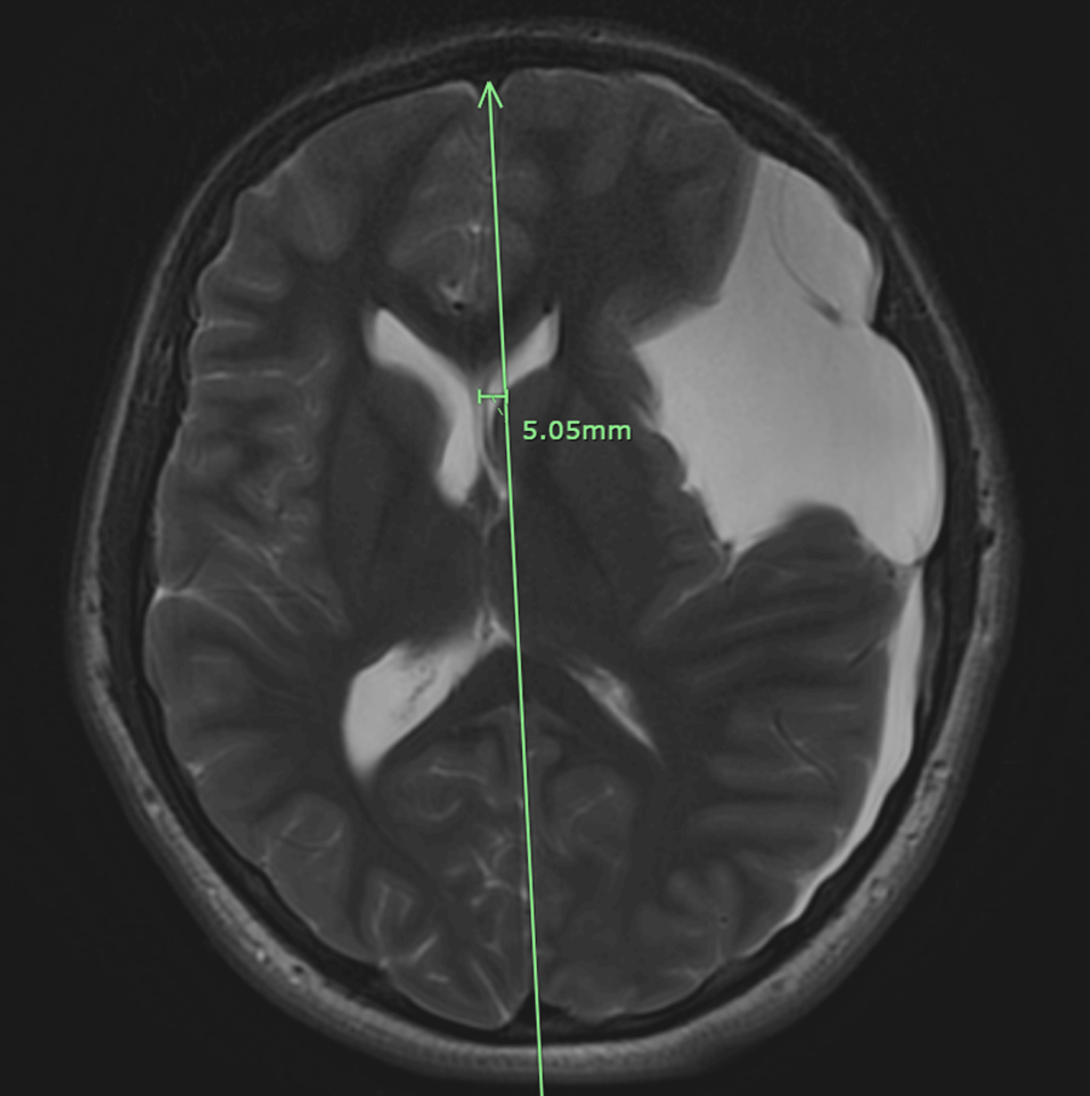
Axial T2-weighted brain MRI without contrast using the PROPELLER technique demonstrating a 5 mm midline shift from the arachnoid cyst. PROPELLER technique: Periodically Rotated Overlapping Parallel Lines with Enhanced Reconstruction

This resulted in substantial mass effect, including sulcal effacement, partial compression of the left lateral ventricle, and a 5 mm midline shift (Figure 4). There was also right temporal horn dilatation, suggesting a risk of possible impending uncal herniation. No blood products were seen, making a hemorrhagic cause less likely, but the radiologist noted that a chronic subdural hematoma could not be completely excluded. The radiographic findings were most consistent with a sizable, likely primary arachnoid cyst.

Differential diagnosis

Before obtaining neuroimaging, the differential diagnosis included post-concussion syndrome, intracranial mass lesion, migraine headache, and adverse reaction to the recent stimulant medication change. Given the chronicity and progression of symptoms, structural pathology needed to be excluded. Although the patient had no family history of migraine, the presence of photophobia and phonophobia warranted consideration of this diagnosis. Post-concussion syndrome was initially favored based on clinical history and symptom pattern, but this remained a diagnosis of exclusion [10]. The patient himself was concerned that the change in his ADHD medication was the trigger for worsening symptoms, which is a plausible consideration but lacked objective findings. Ultimately, MRI findings narrowed the differential to include large arachnoid cyst and chronic subdural hematoma. The absence of blood products on imaging made chronic subdural hematoma unlikely, favoring a diagnosis of an arachnoid cyst causing a midline shift.

Management

Due to the MRI findings of significant midline shift, the patient’s mother was contacted urgently and directed to the local tertiary children’s hospital emergency department. There, the patient was evaluated by neurosurgery and ophthalmology, and he was admitted for observation. Ophthalmology was consulted to evaluate the patient given the proximity of the arachnoid cyst to the ocular structures, and it was found to have mildly elevated intraocular pressures. Despite the worrisome imaging, he remained neurologically intact throughout hospitalization.

Repeat brain imaging the following day showed no progression of the midline shift. Given the absence of neurologic decline during his observation, he was discharged home with plans for neurosurgery follow-up in 48 hours. At the subsequent outpatient neurosurgical evaluation, he was scheduled for placement of a ventriculoperitoneal (VP) shunt. However, serial imaging over the following weeks demonstrated a gradual reduction in the size of the cystic fluid collection and resolution of midline shift. As a result of the improvement in mass effect and midline shift, improvement in his headaches and other symptoms reported on SCAT5, and stable neurologic exam, neurosurgery ultimately deferred shunt placement, and the patient continued conservative, non-operative management. Outpatient ophthalmology follow-up showed decreasing intraocular pressures. This decision reflects the individualized approach to arachnoid cysts, which may not require surgical intervention if the cyst is stable or improving [11]. Ultimately, it was determined by neurosurgery that he had a symptomatic, primary arachnoid cyst that was suspected to have ruptured during the concussion 14 weeks prior to presentation at our office.

Outcome and follow-up

The patient remained under close neurosurgical follow-up for three months. He was restricted from all physical activity during this time. At the end of the observation period, imaging demonstrated continued regression of the cyst and resolution of mass effect. His intraocular pressure also reduced to the normal range.

Based on neurosurgical recommendations, he was cleared to return to physical activity, with the exception of contact sports. Lifelong contact sports restriction was recommended due to residual cyst and the risk of rupture. He has not returned to football or any other high-impact athletic activity since the injury. He remains asymptomatic.

## Discussion

This case highlights the importance of advanced imaging in PCS for both primary care and sports medicine physicians caring for patients with concussions. Persistent post-concussive symptoms should prompt clinicians to consider alternative or concurrent diagnoses beyond post-concussion syndrome. Arachnoid cysts are typically benign, congenital lesions that arise from developmental splitting of the arachnoid membrane [4]. Their prevalence in the pediatric population is estimated at 0.3-1%, and most are discovered incidentally in the pediatric population post-traumatically [12]. While the exact incidence is unknown, it is estimated to be higher than initially thought, likely due to advances in imaging [12]. Arachnoid cysts are often asymptomatic but can become clinically significant if they rupture spontaneously or due to trauma-causing subdural effusion, intracystic hemorrhage, or mass effect [7,8]. Rupture following trauma accounts for approximately 75% of symptomatic cases [8]. Younger age and male sex are considered risk factors, likely due to both biological predisposition and higher exposure to contact sports [1].

Diagnostic challenges

In this case, the patient’s symptoms were initially attributed to PCS as well as a recent stimulant medication change, which distracted from the underlying structural abnormality. This illustrates the cognitive bias of diagnostic anchoring, where early assumptions can delay more thorough investigations. Although he was meeting diagnostic criteria for PCS and recently had a stimulant medication change, it was imperative that we consider structural pathology in our differential diagnoses. The absence of gross neurological deficits may have made the diagnosis of a ruptured arachnoid cyst less likely, but advanced imaging revealed a large lesion with a 5 mm midline shift, a finding that could have progressed to herniation if untreated or he had returned to contact sport.

Management options

Management of arachnoid cysts depends on symptoms and imaging findings. Neurosurgical intervention is generally reserved for cases with evidence of increased intracranial pressure, progressive neurologic deficits, or radiographic progression [6]. In the present case, serial imaging showed regression, and our patient showed a stable neurological exam with a decline in his symptoms prior to placement of his VP shunt, allowing for conservative management without operative intervention. Clinicians should maintain a high index of suspicion for structural lesions in patients with atypical or prolonged concussion recovery [7]. Return to play guidance should involve the athlete, family, team physician, and neurosurgeon. Other factors considered with the decision to return to play include the need for surgical intervention, sport's level of risk, competition level, and neurologic recovery from the initial injury [13]. Ultimately, it was recommended by neurosurgery that our athlete be allowed to participate in non-contact sports only due to the risk of re-injury to the arachnoid cyst.

## Conclusions

Arachnoid cysts are relatively common incidental findings in children but can become symptomatic following trauma. Persistent symptoms after concussion should prompt consideration of structural brain lesions, especially when recovery is atypical. Large arachnoid cysts can result in significant mass effect with potential for herniation. Advanced imaging is essential when post-concussion symptoms persist or worsen, particularly in pediatric contact sport athletes. Conservative management may be appropriate in selected cases of arachnoid cysts showing radiographic improvement and clinical stability. Return to play after structural brain abnormality is a complicated decision and involves the athlete, family, team physician, and neurosurgeon when evaluating the risk of return to sport.
